# Νanomaterial-Loaded Polymer Coating Prevents the In Vitro Growth of *Candida albicans* Biofilms on Silicone Biomaterials

**DOI:** 10.3390/antibiotics12071103

**Published:** 2023-06-25

**Authors:** Alexios Tsikopoulos, Konstantinos Tsikopoulos, Gabriele Meroni, Christoforos Gravalidis, Prodromos Soukouroglou, Athanasios Chatzimoschou, Lorenzo Drago, Stefanos Triaridis, Paraskevi Papaioannidou

**Affiliations:** 1 1st Department of Pharmacology, School of Medicine, Faculty of Health Sciences, Aristotle University of Thessaloniki, 54124 Thessaloniki, Greece; kostastsikop@gmail.com (K.T.); ppap@auth.gr (P.P.); 2 One Health Unit, Department of Biomedical, Surgical and Dental Sciences, School of Medicine, University of Milan, 20133 Milan, Italy; gabriele.meroni@unimi.it; 3 Department of Physics, Aristotle University of Thessaloniki, 54124 Thessaloniki, Greece; cgrava@physics.auth.gr; 4 Laboratory of Microbiology, Hippokration Hospital, 54642 Thessaloniki, Greece; maksouko@yahoo.gr; 5 Lab of Infectious Diseases, Hippokration Hospital, 54642 Thessaloniki, Greece; athanasios.chatzimoschou@gmail.com; 6 Laboratory of Clinical Microbiology & Microbiome, Department of Biomedical Sciences for Health, School of Medicine, University of Milan, 20133 Milan, Italy; lorenzo.drago@unimi.it; 7 1st Department of Otorhinolaryngology-Head and Neck Surgery, AHEPA General Hospital, Aristotle University of Thessaloniki, 54636 Thessaloniki, Greece; triaridis@hotmail.com

**Keywords:** *Candida albicans*, biofilm, prevention, nanomaterials, Al_2_O_3_ nanowires, TiO_2_ nanoparticles, in vitro

## Abstract

Early failure of silicone voice prostheses resulting from fungal colonization and biofilm formation poses a major concern in modern ear nose throat surgery. Therefore, developing new infection prevention techniques to prolong those implants’ survivorship is crucial. We designed an in vitro laboratory study to include nanomaterial-enhanced polymer coating with a plasma spraying technique against *Candida albicans* growth to address this issue. The anti-biofilm effects of high- and low-dose Al_2_O_3_ nanowire and TiO_2_ nanoparticle coatings were studied either alone or in conjunction with each other using checkerboard testing. It was demonstrated that both nanomaterials were capable of preventing fungal biofilm formation regardless of the anti-fungal agent concentration (median absorbance for high-dose Al_2_O_3_-enhanced polymer coating was 0.176 [IQR = 0.207] versus control absorbance of 0.805 [IQR = 0.381], *p* = 0.003 [98% biofilm reduction]; median absorbance for high-dose TiO_2_-enhanced polymer coating was 0.186 [IQR = 0.024] versus control absorbance of 0.766 [IQR = 0.458], *p* < 0.001 [93% biofilm reduction]). Furthermore, synergy was revealed when the Bliss model was applied. According to the findings of this work, it seems that simultaneous consideration of Al_2_O_3_ and TiO_2_ could further increase the existing antibiofilm potential of these nanomaterials and decrease the likelihood of localized toxicity.

## 1. Introduction

Laryngeal cancer poses a significant health and social burden with an average of 3.28 disability-adjusted life years (DALYs) per year [1]. Regrettably, approximately 60% of newly diagnosed patients are classified as stages III or IV (i.e., advanced disease) [2]. Of note, the standard treatment for advanced laryngeal cancer includes a total laryngectomy (TL) combined with radiotherapy (RT) [3]. One of the main sequelae of TL is voice loss, which can be addressed with the insertion of a silicone voice prosthesis into a surgically created tracheoesophageal fistula [4,5]. On that occasion, the material of choice is silicone rubber because of its excellent mechanical and molding properties [6]. However, the hydrophobicity of silicone rubber surfaces [7] in conjunction with the continuous exposure to saliva, food, drinks, and oropharyngeal microflora [8] contribute to rapid microbial colonization of the prosthesis [9] and subsequent biofilm formation [10], thus, resulting in the malfunctioning of this medical device. Therefore, achieving long-term voice restoration is challenging not only because the mode of communication becomes different after TL but also because of the frequent prosthesis replacements secondary to localized infections [10].

From a pathophysiological perspective, it is undeniable that biofilm formation is the primary cause of implant-associated infections [11,12]. In particular, biofilms are complex 3D structures that feature microorganism communities enclosed in a self-synthesized matrix of exopolymeric substances [13]. Those structures serve as barriers to the diffusion of antimicrobial compounds inside the biofilm [14,15]. As a result, biofilms are remarkably resistant not only to antifungal chemotherapy but also to the immune response itself. Thus, inhibiting bacterial adhesion is often regarded as the most critical step in preventing implant-associated infection.

Among the different microorganisms that can easily colonize silicone vocal implants, fungal species are the most common culprits, with a prevalence of 72.9% [11]. It is underlined that the predominant yeasts genera implicated in biofilm formation are *Candida* strains, including but not limited to *Candida albicans*, which is an opportunistic pathogen able to generate not only superficial but also deep-seated infections in immunocompromised patients [16]. From a clinical viewpoint, early signs of biofilm formation in the setting of a vocal implant include escape/leakage of esophageal contents, increased airflow resistance, and thickening of the walls. Usually, the above signs lead to the replacement of an indwelling voice prosthesis [14], thereby limiting its survivorship to 4–6 months [17]. On top of that, the consequences of device infection are not limited to the implant’s viability only, as established biofilms can stimulate an inflammatory response and formation of granulation tissue, which necessitates further surgical interventions [14,17,18].

In the last few decades, various anti-biofilm methods have been described to prolong the device’s lifespan. However, implementing antimycotic or antibiotic agents [19] appears inappropriate given the high risk of drug resistance development [20]. For instance, it has been evidenced that fungi’s metabolism and physiology make them notoriously resistant to chemotherapy [21]. What is more, with the required antifungal doses being up to 1000-times higher [22] than those of the minimum inhibitory concentrations (MICs) of standard chemotherapeutic agents, eradication of established *Candida albicans* biofilms with anti-fungal medication appears to be impossible in clinical practice [19,20,21,22,23]. Therefore, new biofilm prevention techniques are required to increase the durability of those devices and the quality of life for the patients.

To ensure the sustained release of antifungal agents in addition to passive protection stemming from implant surface modification, a combination of active and passive coating could be a viable option taking into account the excellent track record of this modality against fungal-induced infections reported in earlier literature [14]. Implementing nanomaterials (i.e., ultrafine particles made of biocompatible materials) as active coating components appears as a promising avenue to explore, as they tend to enhance implant properties advantageously [24]. Therefore, in the present study, we sought to assess the prevention potential of TiO_2_ nanoparticle and Al_2_O_3_ nanowire coating against *C. albicans* biofilms by using an in vitro model of infection with silicon disks simulating voice prostheses.

## 2. Methods

A clinical strain of *C. albicans* was isolated from a silicon catheter and identification was performed with the Vitek^®^ 2 device (bioMerieux, Paris, France). The microbiological experiments were conducted in Hippokration Hospital, Thessaloniki, Greece (IRB 22049/6-5-2022) and the purely pharmacological investigations were carried out in the 1st Department of Pharmacology, School of Medicine, Faculty of Health Science, Thessaloniki, 54124, Greece. In addition, scanning electron microscopy (SEM) studies were conducted at the Department of Physics, Aristotle University of Thessaloniki, Thessaloniki, Greece.

### 2.1. Biomaterials

Medical-grade silicon sheets were purchased from the commercial industry. Subsequently, disks measuring 6 mm (diameter) by 0.5 mm (thickness) were derived from silicone sheets and sterilized by autoclaving at 121 °C for 15 min. For reproducibility reasons, disks were measured with analytical balance before and after the coating application. The median increase in disk weight was found to be 2.82 mg (IQR 1.25).

### 2.2. MIC Determination

The present study determined minimum inhibitory concentration (MIC_50_) using a broth microdilution assay in line with the Clinical and Laboratory Standards Institute guidelines [25]. MIC was defined as the lowest concentration of the antifungal agent that did not allow for C. albicans growth. In brief, a suspension of C. albicans was recovered from Sabouraud agar plates (Millipore, Paris, France) (Supplemental file S1) to an optical density of 0.7 McFarland (approximately 1.5 × 10^8^ colony-forming units [CFU]/mL) and successively inoculated to a final concentration of 10^5^ CFU/mL in a 96-well microplate containing serial twofold dilutions of the testing molecules. MIC values, corresponding to the lowest concentration exhibiting no visible fungal growth, were read after 48 h of aerobic incubation at 37 °C. The effects of the following compounds were evaluated: (1) TiO_2_ nanoparticles (Nanografi, Ankara, Turkey); (2) Al_2_O_3_ nanowires (diam. × L 2–6 nm × 200–400 nm Sigma Aldrich); (3) Fluconazole (Braun, Melsungen, Germany); Amphotericin B (Pharmazac A.Ε., Athens, Greece). Experiments were conducted in triplicates (technical repetitions) to ensure reliability in the results.

### 2.3. Mature Biofilm Production and Minimum Biofilm Inhibitory Concentration (MBIC) Determination

First, we verified *C. albicans’*s ability to produce mature biofilm by staining the poly-saccharide structure of the extracellular matrix of biofilms with safranin. More precisely, mature biofilms were mechanically rinsed with PBS to remove the free-floating microorganisms. Biofilms were then stained with 200 μL of 0.1% safranin for 5 min, rinsed with water, and after that, the absorbance was spectrophotometrically measured at 492 nm (Epoch^TM^ BioTek, Winooski, VT, USA). After carrying out the above experiment, we could confirm that the clinical *C. albicans* isolates we used were strong biofilm producers.

### 2.4. Scanning Electron Microscopy and Coating Assessment

Scanning electron microscopy (SEM) was implemented (FESEM-JSM-7610 Fplus Thermal, Analytical FE SEM, Japan, Tokyo) with the samples being mounted on bronze substrates with an adhesive double-sided carbon tape. For SEM observations, the above samples were coated with carbon, having an average thickness of 200 Å, using a vacuum evaporator JEOL 4X. In regards to the coating assessment, energy dispersive X-ray spectroscopy was performed and results were graphically presented.

### 2.5. Simultaneous Exposure of Biofilms and Planktonic Cells to Al_2_O_3_ and TiO_2_

Mature biofilms and planktonic cells were incubated separately in a checkerboard format for 24 h at 37 °C in RPMI medium (control) or with serially 2-fold-diluted concentrations of TiO_2_ ranging from 0.015 to 32 mg/L and of Al_2_O_3_ ranging from 64 to 4096 mg/L. The metabolic activity of the biofilm and planktonic cells was then measured using the XTT assay. Biofilm MICs in the presence of different concentrations of Al_2_O_3_ and TiO_2_ alone or in combination were determined.

### 2.6. Synergy Assessment between Al_2_O_3_ and TiO_2_

The synergistic or antagonistic effects between Al_2_O_3_ and TiO_2_ were assessed in line with Bliss’s independence model [26]. For credibility reasons, assays were carried out in 6 replicates on different days. To determine the expected theoretical percentage of growth (*E*_ind_) an agent-free control was used as a reference. Ultimately, the effect of the combination of two agents was calculated with the following equation: *E*_ind_ = *E_A_* × *E_B_*, with *E_A_* and *E_B_* representing the experimental growth percentages when each agent acts alone. More specifically, for each independent replicate experiment, for each combination of *x* mg/L of agent A with *y* mg/L of agent B, the observed percentage of growth (*E*_obs_) was subtracted from *E*_ind_. When the mean ΔΕ (ΔΕ = *E*_ind_ − *E*_obs_) was positive and its 95% confidence interval (CI) did not include 0, significant synergy was claimed for that specific combination of agent A with agent B. When the mean ΔΕ was negative without its CI overlapping 0, statistically significant antagonism was claimed. In any other case, indifference was concluded.

### 2.7. Coating Technique

First of all, we note that low- and high-Al_2_O_3_ and TiO_2_ concentrations were defined as 4× and 16× MIC. For polymer coating, Resomer^®^ (Sigma-Aldrich, Milan, Italy) was utilized. To achieve an even distribution of the coating components, an airbrush spray-coating technique was implemented, which featured an appropriate nozzle to substrate a distance of 20 cm, a suitable nitrogen pressure of 1 bar, and a continuous spraying time of 60 s. First, the substrates were placed in the designated fixed and planar position, and subsequently, the solution was loaded into the reservoir to enable spraying. Particular attention was paid when positioning the airbrush, as a completely vertical orientation allowed the formation of a spraying cone with a radius of ~60 mm. The ejected droplets were then collected and merged over the entire substrate, thus, forming a continuous wet film. The resulting films were left to dry freely in the air without thermal annealing. For reproducibility reasons, a pictorial presentation of our unique coating technique is presented in Supplemental file S2.

In terms of the coating gel composition, nanoparticles were diluted in 2 mL of Dimethyl sulfoxide (DMSO). Then, 10 mL of 90% alcohol was added. Last, 8 mL of water (i.e., for injectable preparation) were added to reach a total of 20 mL of sprayable gel. In addition, coating thickness was quantified by using SEM.

### 2.8. Colorimetric Assessment

Measurement of biofilm or planktonic cell metabolic activities was performed using the XTT metabolic-reduction assay. Briefly, after incubation for 48 h, the plates were centrifuged at 4000 rpm for 30 min. After centrifugation, PBS containing 0.25 mg/mL XTT and 40 μg/mL coenzyme Q_0_ was added. After incubation at 37 °C for 1 h, 100 μL was transferred to a new plate and the optical density (OD) was assessed spectrophotometrically. An automated plate reader measured absorbance at 450 nm. Percent metabolic activity was calculated with the following equation: (1 − *X*/*C*) × 100, where *X* is the OD of agent-containing wells and *C* is the OD of control wells with fungi only.

### 2.9. Statistical Analysis and Interpretation of the Results

Statistical analyses were performed using SPSS 29.0 software (SPSS, Chicago, IL, USA), with the dependent variables being absorbance measurements and the independent ones being the intervention groups. After determining the non-normality of our data using normality and non-normality tests (Shapiro–Wilk, D’Agostino and Pearson test and Kolmogorov-Smirnov test), the comparison of medians between two and multiple groups were achieved using non-parametric tests, including Mann–Whitney and Kruskal–Wallis, respectively. The sample size was calculated in advance of the biofilm experiments according to published guidelines governing in vitro research [27]. The calculation was based on the primary outcome of the present study, which featured a desired biofilm prevention varying between 80 and 100% [28]. With the statistical power set at 0.8 and a and b errors at 5% and 20%, respectively, a minimum of 8 disks per testing condition were determined. Of note, Prism 9 (GraphPad Software, Inc, La Jolla, CA, USA) software was utilized for graph generation and a *p*-value of < 0.05 indicated significance.

### 2.10. Interpretation of the Results

Statistical and clinical relevance were taken into consideration in order to interpret our results clinically. In particular, for a comparison to be clinically relevant, the minimum biofilm prevention threshold of 80% was required to be achieved.

## 3. Results

Among the tested antifungal agents, fluconazole was the most effective against *C. albicans* planktonic form with an MIC of 0.25 μg/mL (Table 1). For the remainder of the antifungal drugs that we tested, Amphotericin MIC was found to be 0.5 μg/mL. For the biofilm assay, Al_2_O_3_ and fluconazole were equally effective at preventing an implant infection in vitro (Table 1).

### 3.1. Synergy Assessment

Effect of simultaneous combination of antifungal treatment on biofilms or planktonic cells.

**Figure 1:**
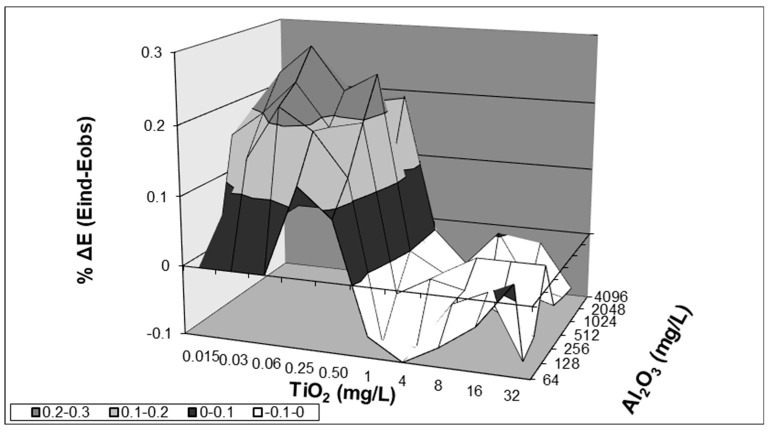
Interaction surface plots obtained from analysis with the Bliss independence model of Al_2_O_3_-TiO_2_ interactions against biofilms of *C. albicans*. Plots represent combinations of Al_2_O_3_ and TiO_2_. The zero plane (ΔΕ = 0) represents indifferent interactions, whereas volumes above (ΔΕ > 0) and below (ΔΕ < 0) the zero plane suggest synergistic and antagonistic interactions, respectively.

Simultaneous treatment of *C. albicans* biofilms with TiO_2_ (0.03 to 0.5 mg/L) and Al_2_O_3_ (128 to 512 mg/L) resulted in synergistic interaction (mean ΔΕ value of significant interactions, 24% (range, 18% to 30%) (Figure 1 and Table 2). In contrast, all combinations of TiO_2_ and Al_2_O_3_ studied exhibited indifferent interactions against planktonic cells (Supplemental file S3).

### 3.2. Impact of Al_2_O_3_- and TiO_2_- Resomer^®^ Coating on Candida Biofilm Growth

**Figure 2:**
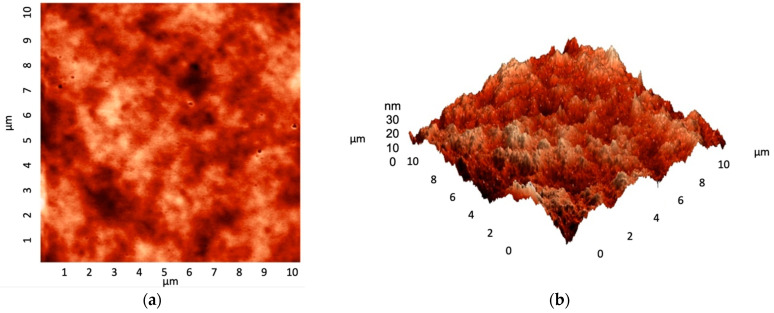
(**a**) 2D and (**b**) 3D atomic force microscopy images of silicon disk surface with calculated roughness of 3.4 nm, respectively.

**Figure 3:**
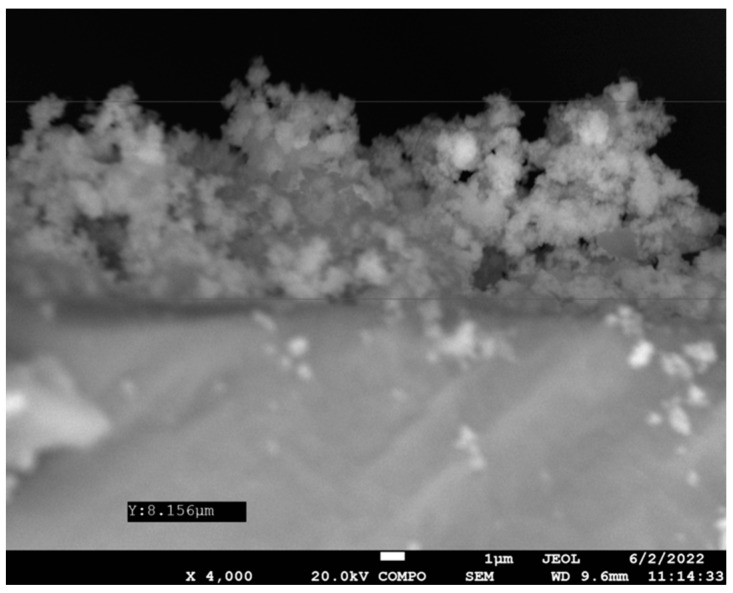
SEM cross-sectional view demonstrating Resomer^®^ coating 8.156 μm thick lying on the top of a silicon disk.

Before studying the results of nanomaterial-impregnated coating on biofilm growth, the roughness of silicone implants was measured, and coating thickness was quantified. In more detail, the roughness was found to be 3.4 Ra with a peak-to-valley height measuring 35.9 nm (Figure 2a,b). In addition, the coating thickness was found to be 8.156 μm (Figure 3).

**Figure 4:**
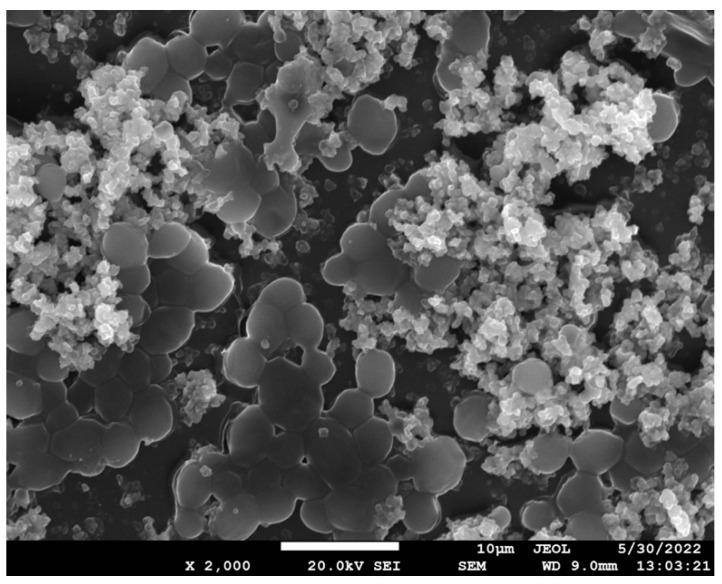
SEM picture depicting *Candida albicans* biofilms (5–9 μm) in conjunction with Resomer^®^ coating on a silicon disk.

After successful coating with 4 × MIC and 16 × MIC for each of the titanium and aluminum implants (Figure 4), disks were placed in 96-well plates and inoculation took place. Following sufficient incubation, disk preparation with mechanical rinsing, vortexing, and sonication, biofilms were appropriately studied, and statistically significant differences were demonstrated relative to positive controls (*p* < 0.05) (Table 3).

**Figure 5:**
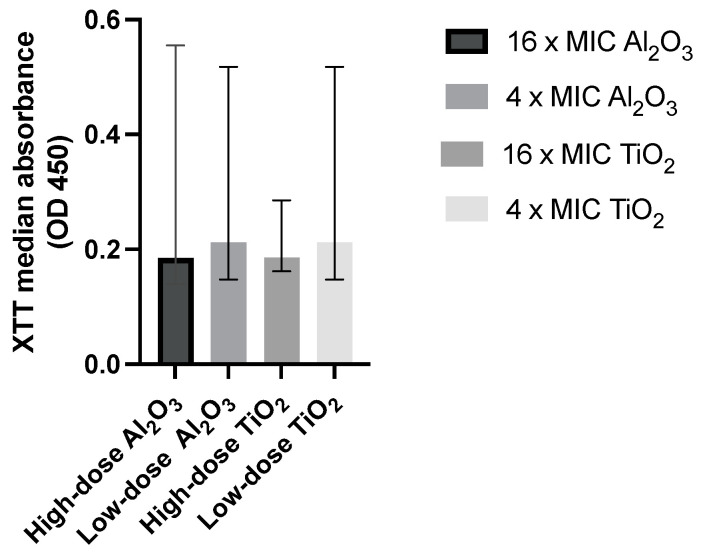
Pictorial presentation of median XTT absorbance relative to actively coated Resomer^®^ groups. No statistically significant difference between groups is demonstrated. MIC = Minimum Inhibitory Concentration; OD = Optical Density; XTT = XTT ([2,3-bis{2-methoxy-4-nitro-5-sulfophenyl}-2*H*-tet- razolium-5-carboxanilide]).

For the between-group analyses, we note that there was no statistically significant difference when we compared absorbance between the intervention groups that featured a combined active and passive coating (i.e., low-dose TiO_2_ vs. high-dose TiO_2_ vs. low-dose Al_2_O_3_ vs. high-dose Al_2_O_3_) (*p* = 0.14) (Figure 5). More specifically, no statistical significance was revealed when low-dose TiO_2_ coating was assessed against high-dose TiO_2_ (*p* = 0.309). Likewise, no difference was demonstrated between low-dose Al_2_O_3_ and high-dose Al_2_O_3_ (*p* = 0.15). When compared to polymer coating alone, the nanomaterial-enhanced coating did not yield any statistically significant absorption differences (*p* > 0.05).

### 3.3. Coating Assessment and Characterization Data

**Figure 6:**
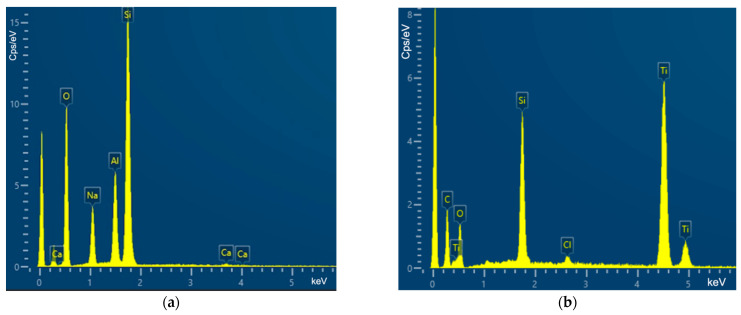
(**a**) Energy dispersive X-ray spectroscopy for Al_2_O_3_ coating assessment on silicone disk. (**b**) Energy dispersive X-ray spectroscopy for TiO_2_ coating assessment on silicone disk.

As per energy dispersive X-ray spectroscopy evaluation, the coating was successfully assessed not only for TiO_2_ nanoparticles but also for Al_2_O_3_ nanowires (Figure 6a,b).

## 4. Discussion 

In the present in vitro study, we demonstrated that not only Al_2_O_3_ nanowire—enhanced but also TiO_2_ nanoparticle—impregnated Resomer^®^ coatings could prevent *C. albicans* growth in the presence of silicone disks simulating ear nose throat implants. Our finding is in keeping with earlier meta-analyses of in vitro literature supporting the fact that combined passive and active coating yields optimal infection-related outcomes for yeast infections [14]. Moreover, the synergy between the above biomaterials was documented when we simultaneously assessed the combined efficacy of those antifungal agents with checkerboard testing. Nevertheless, these promising findings should be interpreted with caution due to the fact that there are differences between in vitro and in vivo behavior, and the results of in vitro studies also need experimental studies before being clinically translated.

Regarding the efficacy of Resomer^®^-supplemented coating, a dose-dependent biofilm inhibition was recorded when we loaded our polymer coating with Al_2_O_3_ nanowires and TiO_2_ nanoparticles. To elaborate further, we note that although all tested concentrations exceeded the clinically meaningful biofilm inhibition threshold, high-dose Al_2_O_3_ coating could inhibit more than 95% of *C. albicans* biofilm formation. However, using large concentrations of nanomaterials may result in local and/or systemic toxicity, which in turn raises safety issues. To mitigate this toxicity risk, combining nanomaterials could be a great avenue to explore. Interestingly enough, indifference was demonstrated when we combined TiO_2_ and Al_2_O_3_ against the planktonic form of *C. albicans*. By contrast, the synergy between the above nanomaterials was revealed when the Bliss model was implemented for biofilm form. Therefore, we advocate that a combination of between Al_2_O_3_ nanowire- and TiO_2_ nanoparticle-coating may yield better silicone device protection while maintaining anti-fungal agents’ concentrations at lower levels. Likewise, recent animal research has suggested that synergistic antibacterial effects against Staphylococcus aureus are exhibited when metallic nanomaterials are combined [29].

### 4.1. Toxicity Concerns

It should be mentioned that nanotoxicity concerns have been raised by earlier authors, who concluded that nanoparticles are potentially dangerous for human beings depending on their nature, size, surface area, shape, aspect ratio, crystallinity, dissolution, and agglomeration [30,31]. When it comes to assessing toxicity, we wish to underline that nanowire coating is potentially more advantageous than its nanoparticle counterpart. This is because recent in vitro evidence has suggested that utilizing Al_2_O_3_ nanowires results in significantly less toxicity compared to using Al_2_O_3_ nanoparticles [30,32]. On top of that, recent animal evidence has shown that bone toxicity is proportional to increased Al_2_O_3_ nanomaterial concentrations in coatings. Therefore, we suggest that clinicians consider the payoff between toxicity and cost when selecting nanomaterials for clinical use. 

### 4.2. Coating Remarks

Al_2_O_3_ nanowires and TiO_2_ nanoparticles were added to the Resomer^®^ first because they had never been studied in this environment before in the literature. Moreover, although fluconazole was equally effective at preventing *Candida* biofilm growth, this drug was not considered a coating option due to the potential for resistance development.

Furthermore, we wish to highlight the importance of the coating technique when it comes to standardizing the application of bioresorbable material on silicone implants. To be more exact, we claim that ensuring that even distribution of coating components on the biomaterials provides more predictable protection against fungal biofilm growth. To reflect on the above, we verified the formation of a continuous coating layer by using SEM. The present study applied the coating on a smooth silicon surface as the Ra value was less than 10 (ISO 14607, Corrected version 2018-08). More importantly, a long-lasting coating effect on silicone implants was recorded, providing sustained protection against fungal growth.

### 4.3. Coating Considerations

Recently published evidence has suggested that coating is a complex phenomenon which cannot be successfully determined using a single statistical test [33]. In other words, the bonding strength is correlated not only with surface roughness but also with chemical bonds, surface cleanliness, and mechanical factors. However, it has been postulated that achieving a desired controlled surface roughness can effectively decrease the shear forces exerted on the coating components, thus, enhancing its adhesion properties [34].

### 4.4. Study Limitations and Implications for Future Research

We recognize that the present in vitro study has a few limitations. First, although the present in vitro investigation results were promising, we wish to draw the readers’ attention to the fact that unwarranted extrapolations to human biology should be avoided. In other words, a stepwise research approach is required to confirm results in living organisms, including conducting a small animal model study. This may be followed by large animal model preclinical investigations provided that the clinical results remain satisfactory. Additionally, we wish to underline that there are significant differences between fungal growth in the lab setting compared to real-life. Therefore, we claim that future research could also focus on ex vivo models of infection, as those models appear to be advantageous over their in vitro counterparts given the fact they maintain crucial biological factors from the hosts [33,35].

Second, although we observed a long-lasting effect of Resomer^®^ coating in the lab over the course of two weeks, we advocate that local mechanical factors may affect its characteristics in a complex in vivo environment. To elaborate, continuous exposure of coated silicone implants to food and saliva may harm coating integrity and longevity in the pharyngeal environment. Future studies could concentrate on more durable implant surface finishing techniques to ensure long-term device protection from fungi. Alternatively, in order to avoid costly surface modifications, the application of an additional layer above the one of PDLLA may yield a more durable effect while maintaining the cost at low levels. On top of that, it should be underlined that determining the adhesion properties of the suggested coating method is important in clinical settings, and, therefore, conducting further investigation on this matter should be prioritized by future authors in this field.

Third, given the synergistic effects against *Candida* biofilm growth, further research is needed to identify the optimal combination between TiO_2_ nanoparticle and Al_2_O_3_ nanowire-coating to not only to optimize the infection prevention potential but also minimize side effects and cytotoxicity. For a thorough evaluation, not only localized but also systemic toxicity (that is, impact on renal, liver, and lung cells) should be investigated in future papers.

## 5. Conclusions

In this in vitro study, we investigated the antifungal properties of TiO_2_ nanoparticles and Al_2_O_3_ nanowires against *Candida albicans* and promising results were demonstrated regardless of the coating concentration we implemented. In particular, a standalone application of TiO_2_ nanoparticle and Al_2_O_3_ nanowire Resomer^®^ coatings yielded greater than 85% reduction of *Candida albicans* biofilm growth on silicone disks in our in vitro model of infection. Moreover, the synergy between the mentioned nanomaterials was shown, which could be highly beneficial when considering coatings consisting of multiple components. However, given the novelty of our findings, further research is needed to finetune the coating characteristics and verify results in animal infection models.

## Figures and Tables

**Table 1:** Inhibitory effects of anti-fungal agents against planktonic and biofilm forms of *C. albicans.*

Antifungal Agent	MIC (μg/mL)	MBIC (μg/mL)
TiO_2_ nanoparticles	1024	4096
Al_2_O_3_ nanowires	512	2048
Fluconazole	0.25	2048

MBIC = Minimum Biofilm Inhibitory Concentration; MIC = Minimum Inhibitory Concentration.

**Table 2:** Synergistic effects between AlO_3_ and TiO_2_ against biofilm development as per Bliss’s model.

Al_2_O_3_	TiO_2_									
0	0.015 mg/L	0.03 mg/L	0.06 mg/L	0.25 mg/L	0.50 mg/L	1 mg/L	4 mg/L	8 mg/L	16 mg/L	32 mg/L
64 mg/L	IND	IND	IND	IND	SYN	IND	IND	IND	IND	IND
128 mg/L	IND	SYN	SYN	SYN	SYN	IND	IND	IND	IND	IND
256 mg/L	SYN	SYN	SYN	SYN	SYN	IND	IND	IND	IND	IND
512 mg/L	SYN	SYN	SYN	SYN	SYN	IND	IND	IND	IND	IND
1024 mg/L	IND	IND	IND	IND	IND	IND	IND	SYN	SYN	IND
2048 mg/L	IND	IND	IND	IND	IND	IND	IND	IND	IND	IND
4096 mg/L	IND	IND	IND	IND	IND	IND	IND	IND	IND	IND

SYN = Synergism; IND = Indifference.

**Table 3:** Antibiofilm activity of Resomer^®^ coating considered either alone or supplemented with nanomaterials.

Treatment Group	Median Absorbance (IQR)		*p* Value	% Biofilm Reduction
	Intervention Group	Biofilm Control Group		
High-dose Al_2_O_3_-enhanced polymer coating	0.176 (0.207)	0.805 (0.381)	0.003	98%
Low-dose Al_2_O_3_-enhanced polymer coating	0.25 (0.161)	0.805 (0.381)	0.002	87%
High-dose TiO_2_-enhanced polymer coating	0.186 (0.024)	0.766 (0.458)	<0.001	93%
Low-dose TiO_2_-enhanced polymer coating	0.213 (0.152)	0.766 (0.458)	<0.001	89%
Polymer coating alone	0.246 (0.098)	0.766 (0.458)	<0.001	84%

IQR = interquartile range; *p* < 0.05 indicates statistical significance. High-dose = 16 × MIC; Low-dose = 4 × MIC.

## Data Availability

The raw/processed data required to reproduce these findings cannot be shared at this time due to technical or time limitations.

## Supplementary Materials

The following supporting information can be downloaded at: https://www.mdpi.com/article/10.3390/antibiotics12071103/s1, Supplemental file S1: *Candida albicans* growth on a Sabouraud agar plate. Supplemental file S2: Pictorial presentation of spraying device to achieve even coating of nanomaterial coating of silicon implants. Supplemental file S3: No synergy is demonstrated between Al_2_O_3_ and TiO_2_ nanomaterials against *Candida albicans*.
