# Supplementary material for: Νanomaterial-Loaded Polymer Coating Prevents the In Vitro Growth of Candida albicans Biofilms on Silicone Biomaterials
Source: Antibiotics (Basel). 2023 Jun 25;12(7):1103. doi: 10.3390/antibiotics12071103 (PMC10376674; doi:10.3390/antibiotics12071103)

Supplemental file 2. Pictorial presentation of spraying device to achieve even coating of nanomaterial coating of silicon implants

Sterile vials with a maximum capacity of 10ml

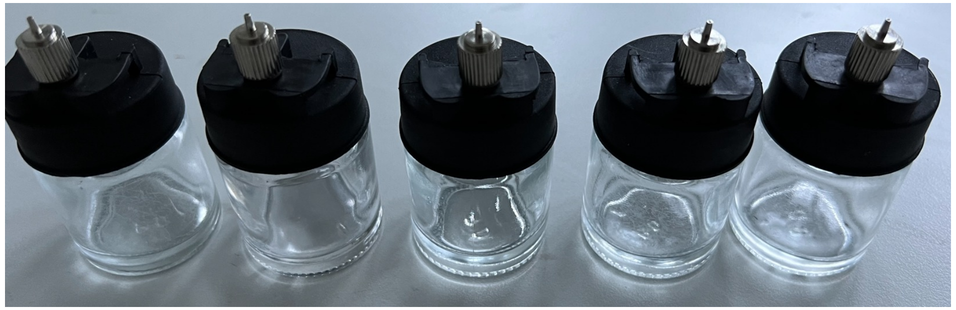

Laser-assisted pointing system for accurate coating implementation

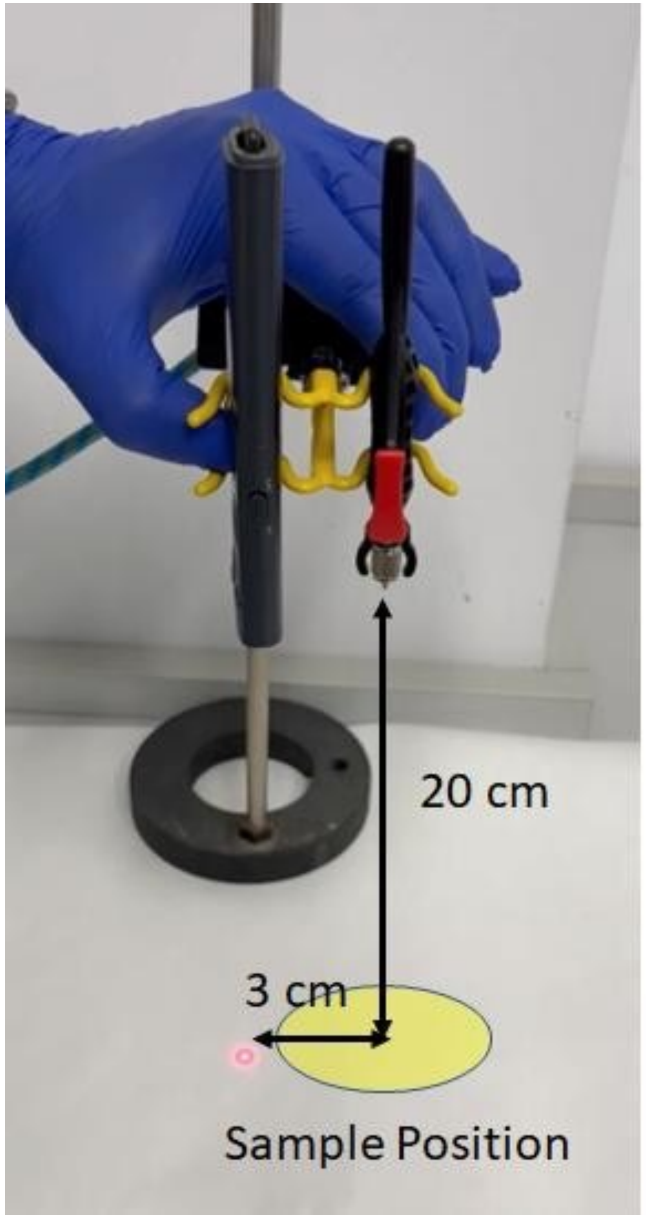

Final position/mounting of spraying device prior to coating application

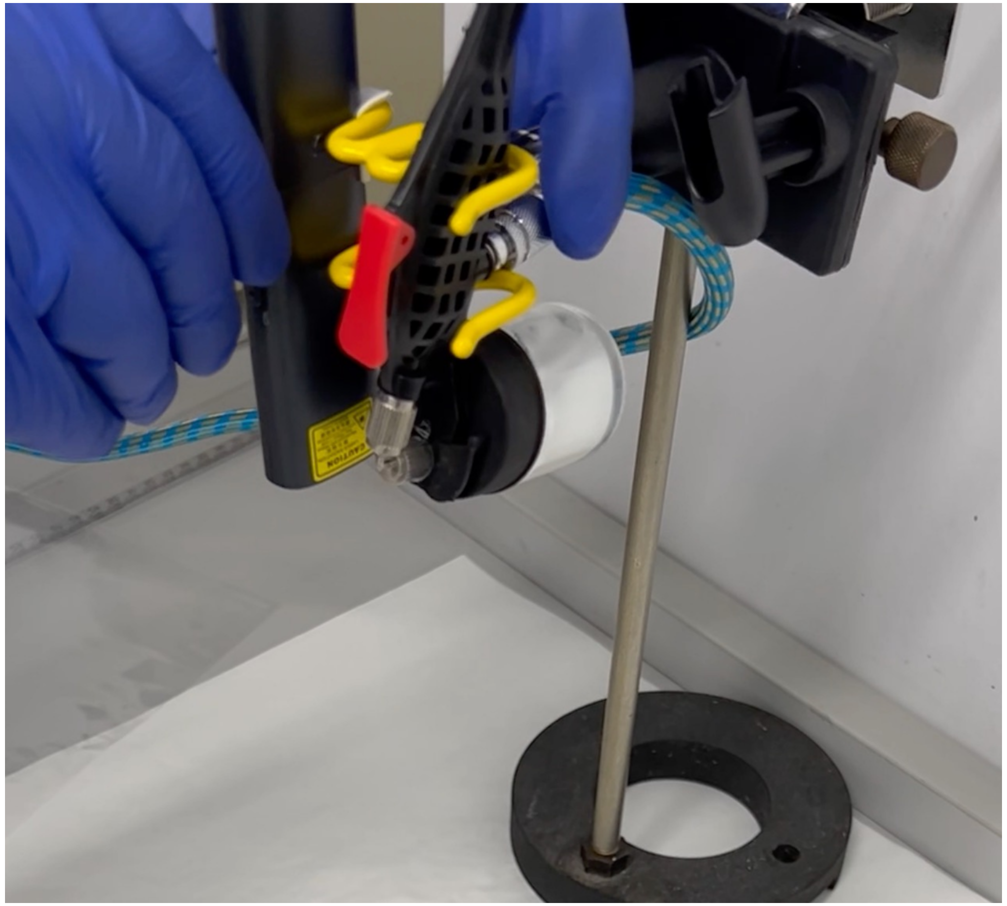

At the conclusion of the coating process, the Resomer® coated silicone disk is positioned in a 96 well plate

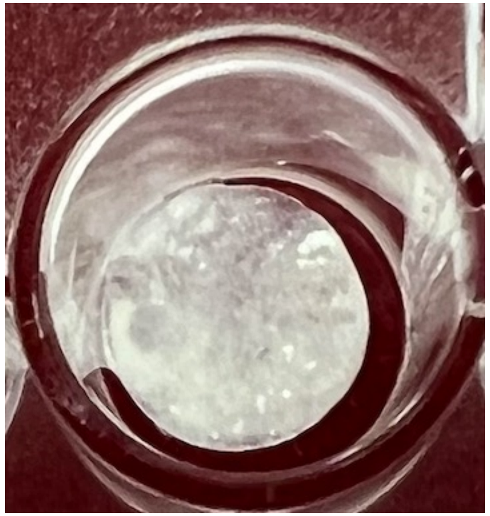

Supplement: Supplementary file 1 [file antibiotics-12-01103-s001.zip › Supplemental file S2.pdf]
