# Supplementary material for: Νanomaterial-Loaded Polymer Coating Prevents the In Vitro Growth of Candida albicans Biofilms on Silicone Biomaterials
Source: Antibiotics (Basel). 2023 Jun 25;12(7):1103. doi: 10.3390/antibiotics12071103 (PMC10376674; doi:10.3390/antibiotics12071103)
Supplement: Supplementary file 1 [file antibiotics-12-01103-s001.zip › Supplemental file S3.pdf]

Supplemental file 3. No synergy is demonstrated between Al_2O_3 and TiO_2 nanomaterials against Candida Albicans

Al2O3	TiO2									
0	0.008 mg/L	0.015 mg/L	0.03 mg/L	0.06 mg/L	0.25 mg/L	0.50 mg/L	1 mg/L	4 mg/L	8 mg/L	16 mg/L
16 mg/L	IND	IND	IND	IND	IND	IND	IND	IND	IND	IND
32 mg/L	IND	IND	IND	IND	IND	IND	IND	IND	IND	IND
64 mg/L	IND	IND	IND	IND	IND	IND	IND	IND	IND	IND
128 mg/L	IND	IND	IND	IND	IND	IND	IND	IND	IND	IND
256 mg/L	IND	IND	IND	IND	IND	IND	IND	IND	IND	IND
512 mg/L	IND	IND	IND	IND	IND	IND	IND	IND	IND	IND
1024 mg/L	IND	IND	IND	IND	IND	IND	IND	IND	IND	IND
