# Supplementary figures and images for: Νanomaterial-Loaded Polymer Coating Prevents the In Vitro Growth of Candida albicans Biofilms on Silicone Biomaterials
Source: Antibiotics (Basel). 2023 Jun 25;12(7):1103. doi: 10.3390/antibiotics12071103 (PMC10376674; doi:10.3390/antibiotics12071103)

**Supplemental file 1.** *Candida albicans* growth on a Sabouraud agar plate

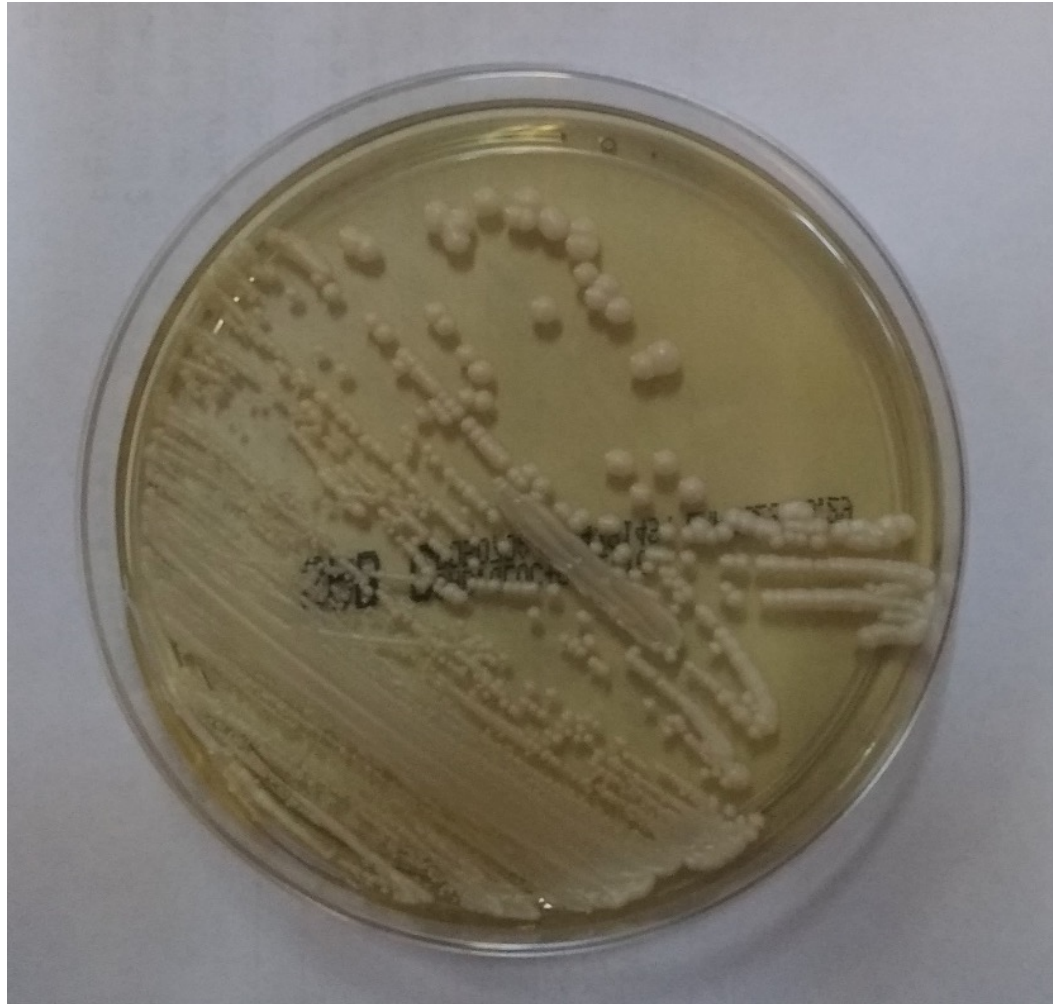

Supplement: Supplementary file 1 [file antibiotics-12-01103-s001.zip › Supplemental file S1.pdf]
